# A SERM increasing the expression of the osteoblastogenesis and mineralization-related proteins and improving quality of bone tissue in an experimental model of osteoporosis

**DOI:** 10.1590/1678-7757-2017-0329

**Published:** 2018-04-18

**Authors:** Fernanda Costa Yogui, Gustavo Antonio Correa Momesso, Leonardo Perez Faverani, Tarik Ocon Braga Polo, Gabriel Ramalho-Ferreira, Jaqueline Suemi Hassumi, Ana Cláudia Rossi, Alexandre Rodrigues Freire, Felippe Bevilacqua Prado, Roberta Okamoto

**Affiliations:** 1 Universidade Estadual Paulista (UNESP), Faculdade de Odontologia de Araçatuba, Departamento de Ciências Básicas, Araçatuba, São Paulo, Brasil.; 2 Universidade Estadual Paulista (UNESP), Faculdade de Odontologia de Araçatuba, Departamento de Cirurgia e Clínica Integrada, Araçatuba, São Paulo, Brasil.; 3 Universidade Estadual de Campinas, Faculdade de Odontologia de Piracicaba, Departamento de Anatomia, Piracicaba, SP, Brasil.

**Keywords:** Raloxifene, Immunohistochemistry, Osteoporosis, Dental implants, WNT signaling

## Abstract

Raloxifene is an antiresorptive drug, selective estrogen receptor modulator (SERM) used in the treatment of osteoporosis.

**Objective:**

To evaluate proteins related to bone repair at the peri-implant bone in a rat model of osteoporosis treated with raloxifene.

**Material and Methods:**

72 rats were divided into three groups: SHAM (healthy animals), OVX (ovariectomized animals), and RLX (ovariectomized animals treated with raloxifene). Raloxifene was administered by gavage (1 mg/kg/day). Tibial implantation was performed 30 days after ovariectomy, and animals were euthanized at 14, 42, and 60 days postoperatively. Samples were collected and analyzed by immunohistochemical reactions, molecular analysis, and microtomographic parameters.

**Results:**

RLX showed intense staining of all investigated proteins at both time points except for RUNX2. These results were similar to SHAM and opposite to OVX, showing mild staining. The PCR gene expression of OC and ALP values for RLX (P<0.05) followed by SHAM and OVX groups. For BSP data, the highest expression was observed in the RLX groups and the lowest expression was observed in the OVX groups (P<0.05). For RUNX2 data, RLX and SHAM groups showed greater values compared to OVX (P<0.05). At 60 days postoperatively, microtomography parameters, related to closed porosity, showed higher values for (Po.N), (Po.V), and (Po) in RLX and SHAM groups, whereas OVX groups showed lower results (P<0.05); (BV) values (P=0.009); regarding total porosity (Po.tot), RLX group had statistically significant lower values than OVX and SHAM groups (P=0.009). Regarding the open porosity (Po.V and Po), the SHAM group presented the highest values, followed by OVX and RLX groups (P<0.05). The Structural Model Index (SMI), RLX group showed a value closer to zero than SHAM group (P<0.05).

**Conclusions:**

Raloxifene had a positive effect on the expression of osteoblastogenesis/mineralization-related proteins and on micro-CT parameters related to peri-implant bone healing.

## Introduction

Post-menopausal osteoporosis is the main skeletal disorder with high incidence found in society, being characterized by reduced bone mass due to estrogen deficiency and decreased intestinal absorption of calcium, resulting in increased bone fragility and fracture susceptibility^19^. Studies evidenced that one of the signaling pathways of these proteins, as the canonical pathway, has very importance in the knowledge of osteoporosis and drug action used in their treatment^2^.

Many drug therapies were developed for osteoporosis treatment. Among them, we highlight raloxifene, a second generation Selective Estrogen Receptor Modulator (SERM) approved for use in the prevention and treatment of osteoporosis in postmenopausal women. Raloxifene acts by mimicking estrogen actions in the bone tissue and cholesterol metabolism (reducing total cholesterol and LDL levels), playing a role in the regulation of osteoblast lineage cells and bone remodeling^5^ ^,^ ^15^.

Bisphosphonates are the most commonly used drugs for osteoporosis treatment, mainly alendronate, and have shown great results by increasing bone mineral density and decreasing the risk of bone fracture in post-menopausal women^29^ However, the long-term use of these drugs is associated with the emergence of medication-related osteonecrosis of the jaw (MRONJ), increasingly common in patients who underwent oral surgeries, such as implants placement^21^.

The activity of non-collagenous bone matrix proteins synthesized by osteoblasts during the calcium deposition process, such as osteocalcin, osteopontin, and bone sialoprotein, is important in the process of bone matrix maturation and mineralization, and regulates the functional activity of bone cells^10^. Osteocalcin is an important marker of bone mineralization, representing the final stage of bone formation and demonstrating its importance for better understanding bone remodeling^13^.

Clinical studies have demonstrated the positive effect of raloxifene by preventing bone loss and decreasing the risk of bone fracture in post-menopausal women, besides having no relationship with breast cancer^4^ ^,^ ^5^. Furthermore, pre-clinical studies have shown the positive role of raloxifene in the alveolar bone^14^ ^,^ ^15^. However, few *in vivo* studies showed the effect of this drug on osteoblastogenesis proteins during peri-implant healing.

The reason for choosing raloxifene in peri-implant healing of osteoporosis conditions instead of other drugs, such as bisphosphonates, is because this drug is the only one to present a nearly physiologic effect, acting on estrogen receptors. Besides that, this drug can inhibit bone resorption and promote bone formation. On the other hand, bisphosphonates are described as antiresorptive drugs which, in the long-term, can damage bone turnover, inhibit bone resorption, and do not promote bone formation, prevailing a poor quality and old bone, in addition to be related with medication-related osteonecrosis of the jaw (MRONJ).

Thus, this study aimed to evaluate new proteins belonging to the WNT/β-catenin pathway and other proteins related to bone repair and morphometric parameters at the bone-implant interface in induced- osteoporosis rat model treated with raloxifene.

## Material and Methods

### Animals

This study followed ethical principles and was approved by the Ethics Committee on Animal Use of FOA-UNESP under the protocol number 2012/01096. For this study, 72 4-month-old rats *(Rattus norvegicus albinus,* Wistar), weighing approximately 250 grams, were obtained from the central vivarium of FOA-UNESP. The animals were divided into three groups: SHAM - rats submitted to sham surgery and fed a balanced diet; OVX - rats submitted to bilateral ovariectomy and fed a low calcium diet (osteoporotic) without medical treatment; RLX - rats submitted to bilateral ovariectomy and fed a low calcium diet (osteoporotic) with raloxifene treatment.

The animals were kept in cages and fed a balanced diet (NUVILAB, Curitiba, PR, Brazil) containing 1.4% Ca^++^ and 0.8% P, and given water *ad libitum.* After completion of sham surgeries and ovariectomies, animals in the SHAM group continued to receive rations of the balanced diet, whereas animals in the other two groups were switched to a diet containing 0.1% Ca^++^ and 0.5% P (RHOSTER Ind. Com., Vargem Grande Paulista, SP, Brazil).

### Estrous cycle classification

The rats were placed in individual cages for daily observation of estrous cycle. The technique used was described by Long and Evans^8^ (1922) and consisted of introducing 1-2 drops of physiological serum into the vagina, which was then aspirated and placed on a histology slide for microscopic visualization. After observation of two or three regular cycles, animals were selected.

### Induction of osteoporosis

Osteoporosis was induced in animals by using a combination of bilateral ovariectomy and 4-week treatment with a low calcium and phosphate diet, as previously described by Teófilo, et al.^26^ (2004) and Ramalho-Ferreira, et al.^18^ (2016). The development of osteoporosis was confirmed by obtaining cortical bone mineral density (BMD) values from SHAM and OVX rats using computerized micro-tomography (SkyScan 1176, Bruker MicroCT, Aartse-Laar, Belgium). We confirmed that BMD in animals not subjected to ovariectomy and who received a normal diet was 0.35255 g/cm^3^, as compared to 0.12525 g/cm^3^ in ovariectomized animals. This fact confirmed the presence of osteopenia in the latter group, which is characteristic of the osteoporotic rat model.

### Bilateral ovariectomy

RLX and OVX rats were anesthetized, and incisions in both flanks were subsequently performed to expose the ovaries, which were then surgically removed. Rats from the SHAM group were subjected to the same procedure. However, the ovaries were exposed and not removed.

### Drug treatment

Raloxifene treatment (1 mg/kg/day) began eight days after ovariectomy by oral gavage^15^ of aqueous solution containing the drug dissolved. The course of treatment lasted until the end of the experiment (euthanasia of animals), totaling 44, 72, and 90 days of oral administration in RLX and OVX groups, according to the periods of analysis.

### Tibia implantation surgery

Thirty days after drug treatment, the animals were fasted for eight hours prior to the surgery, and were sedated using a combination of 50 mg/kg intramuscular ketamine (Vetaset, Fort Dodge Animal Health Ltd, Campinas, SP, Brazil) and 5 mg/kg xylazine hydrochloride (Dopaser, Laboratory Calier of Brazil Ltda., Osasco, SP, Brazil). Rats received mepivacaine hydrochloride (0.3 ml/kg) and scandicaine (2%) with epinephrine (1:100,000) (Septodont, France) for local anesthesia and the maintenance of operative field hemostasis.

After animals were sedated, a trichotomy was performed in the medial portion of the left and right tibias, and antisepsis of the incision region was performed using polyvinylpyrrolidone iodine disinfectant (10% povidone, Riodeine Degermante, Rioquímica, São José do Rio Preto, SP, Brazil). An incision was then made with a number 15 blade (Feather Industries Ltd., Tokyo, Japan) in the left and right tibial metaphysis regions, followed by divulsion of the soft tissue to expose the bone for implantation.

One hundred-eight commercially grade for titanium double acid-etched surface implants (cpTi, Implalife Biotechnology, Jales, SP, Brazil) were installed in the left and right tibias of the rats. The implants measured 2.0 mm in diameter and 4.0 mm of long square-edge module. A milling was performed with a 1.4 mm diameter spiral cutter mounted on an electric motor (BLM 600; Driller, São Paulo, SP, Brazil) at 1000 rpm, with 0,9% isotonic sodium chloride irrigation (Physiological; Biosintética Laboratories Inc., Ribeirão Preto, SP, Brazil). It was done a manually installation with a square digital key.

Immediately at the end of the surgical procedure, the tissues were relocated and sutured with a 4-0 vicryl thread (Ethicon, Johnson & Johnson, São José dos Campos, SP, Brazil) in a deep plane and with a monofilament thread (nylon 5-0; Ethicon, Johnson & Johnson) for the skin plane. All the animals received a Pentabiotic injection (0.1 ml/kg; Fort Dodge Saúde Animal Ltda, Campinas, SP, Brazil) during the immediate postoperative period.

### Decalcified tissues

Euthanasia was performed at 14 (n = 6) and 42 days (n = 6), with the left and right tibiae removed and immediately fixed in 10% buffered formaldehyde solution (Reagents Analytical, Dental-Hospital Dynamics Ltd., Catanduva, SP, Brazil) for 48 hours, and then soaked in water for 24 hours. The tissue was then decalcified in 10% EDTA solution for 6 weeks and then dehydrated using a gradually increasing alcohol solution gradient. Diaphanization was performed with xylene, and then the samples were finally embedded in paraffin. The tissue block was cut at a thickness of 5 µm using a microtome, and sections were mounted on slides for subsequent immunohistochemical analysis.

### Calcified tissues

Euthanasia was performed at 60 days (n = 6) with the left and right tibiae removed and immediately fixed in 10% buffered formaldehyde solution (Sigma-Aldrich, St. Louis, MO, USA) for 48 hours, then soaked in water for 24 hours and subsequently stored in 70% ethanol until scanning X-ray beam analysis using a digital computed micro-tomography system.

### Immunohistochemical analysis

For immunohistochemistry analysis, polyclonal goat antibodies (Santa Cruz Biotechnology, Dallas, Texas, EUA) were used as primary antibodies against Runt- related transcription factor 2 (RUNX-2; SC-8566), osteopontin (OPN; SC-10591), and osteocalcin (OCN; SC-365797) to characterize the osteoblastic phenotype, as well as beta-catenin (β-catenin; SC-59737) and WNT-10a pathway (Santa Cruz Biotechnology - SC- 6280, Dallas, Texas, EUA), proteins responsible for stem cell differentiation in bone tissue formed during osseointegration.

Immunostaining was visualized using the indirect immunoperoxidase detection method. Blocking of non-specific reactions was performed through the inactivation of endogenous peroxidase using a solution of 3% hydrogen peroxide (Merck, Kenilworth, NJ, USA), 1% bovine serum albumin (Sigma-Aldrich, St. Louis, MO, USA), and 20% of skim milk powder. Antigen retrieval was achieved using citrate phosphate buffer (pH 6.0) in the presence of moist heat.

The secondary antibody used was a biotinylated goat antibody produced in rabbit (Pierce Biotechnology, Rockford, IL, USA), which was treated with biotin and streptavidin (Dako, Glostrup, Denmark), Elite Kit, Avidin and Biotin (Vector Laboratories, Burlingame, Ca, EUA). Diaminobenzidine (Dako, Glostrup, Denmark) was used as the chromogen. Counterstaining was performed with Harris hematoxylin.

Staining was evaluated through a semi-quantitative analysis - the assignation of different “scores”^17^ to the presence of immunostained cells in the repaired region of the peri-implant bone. Analysis was performed using light microscopy (LeicaR DMLB, Heerbrugg, Switzerland), and assigned scores represented: no staining (0), mild staining (1), moderate staining (2), and intense staining (3). Higher scores reflected an increased area of diaminobenzidine-stained cells. The scores of the evaluator were subjected to Kappa test, in which the index was adjusted to >0.8, indicating that the observed values were consistent. Absence of immunostaining was observed when the primary antibody was substituted by the serum of the host species, acting as a negative control for the secondary antibody.

### PCR analysis

Implants were removed through reverse torque and the peri-implant tissue was collected, washed in phosphate buffered saline, and stored in liquid nitrogen. Total RNA was extracted with Trizol reagent (Promega Corporation, Madison, WI, USA) and converted into cDNA (Life kit; Life Technologies, Invitrogen, Carslbad, CA, USA). Real time PCR was performed with the Step One Plus (Applied Biosystems, Waltham, MA USA) using SYBR Green (Applied Biosystems, Waltham, MA, USA). Beta-actin and beta-2 microglobulin (Life Biotechnologies, Invitrogen, Carlsbad, CA, USA) were used for normalization of RUNX2, BSP, ALP, and OC expression by Delta CT method.

### Micro CT - description of two-dimensional (2D) and three-dimensional (3D) morphometric calculations

The parameters used in this study were adopted in accordance with the guide for evaluation of bone microarchitecture in rodents using micro computed tomography.^1^ CTAnalyzer software (Skyscan, Leuven, Belgium) was used to perform 2D and 3D morphometry. Standardization of the region of interest (ROI) was performed by demarcating a rectangular area in the central slice of the implant that occupied a region corresponding to the valleys between the 3^rd^ and 5^th^ threads of the implant. The rectangular area was approximately 0.5 mm in length and 0.8 mm in width.

From that first demarcation, two new rectangular areas occupying the same regions of the implant were demarcated at 50 slices in the proximal and distal directions relative to the bone where the implant was installed, thus a total volume of 100 slices (874.3 µm) was examined.

After ROI standardization, the sequence of images was converted to grayscale by using a scale ranging from 0 to 255, with a minimum value of 70 and a maximum value of 100 in all groups. These amounts were determined based on the visualization of the cancellous bone structure, located in the region of interest.

Micro-tomography parameters analyzed were bone volume (BV), porosity parameters (Po.N; Po.V and Po) and Structural Model Index (SMI), which evaluates the trabecular geometry.

### Statistical analysis

Regarding the micro-CT analysis, all data were initially subjected to homoscedasticity test (Shapiro- Wilk). BV and Po-tot parameters were subjected to Kruskal-Wallis non-parametric test. A P value <0.05 was interpreted as statistically significant. Structural Model Index (SMI) parameter showed non-parametric data under Kruskal-Wallis test. Thus, it was applied a Tukey post-test and all values in which P<0.05 were considered statistically significant.

## Results

### Immunohistochemical analysis

**Figure 1:**
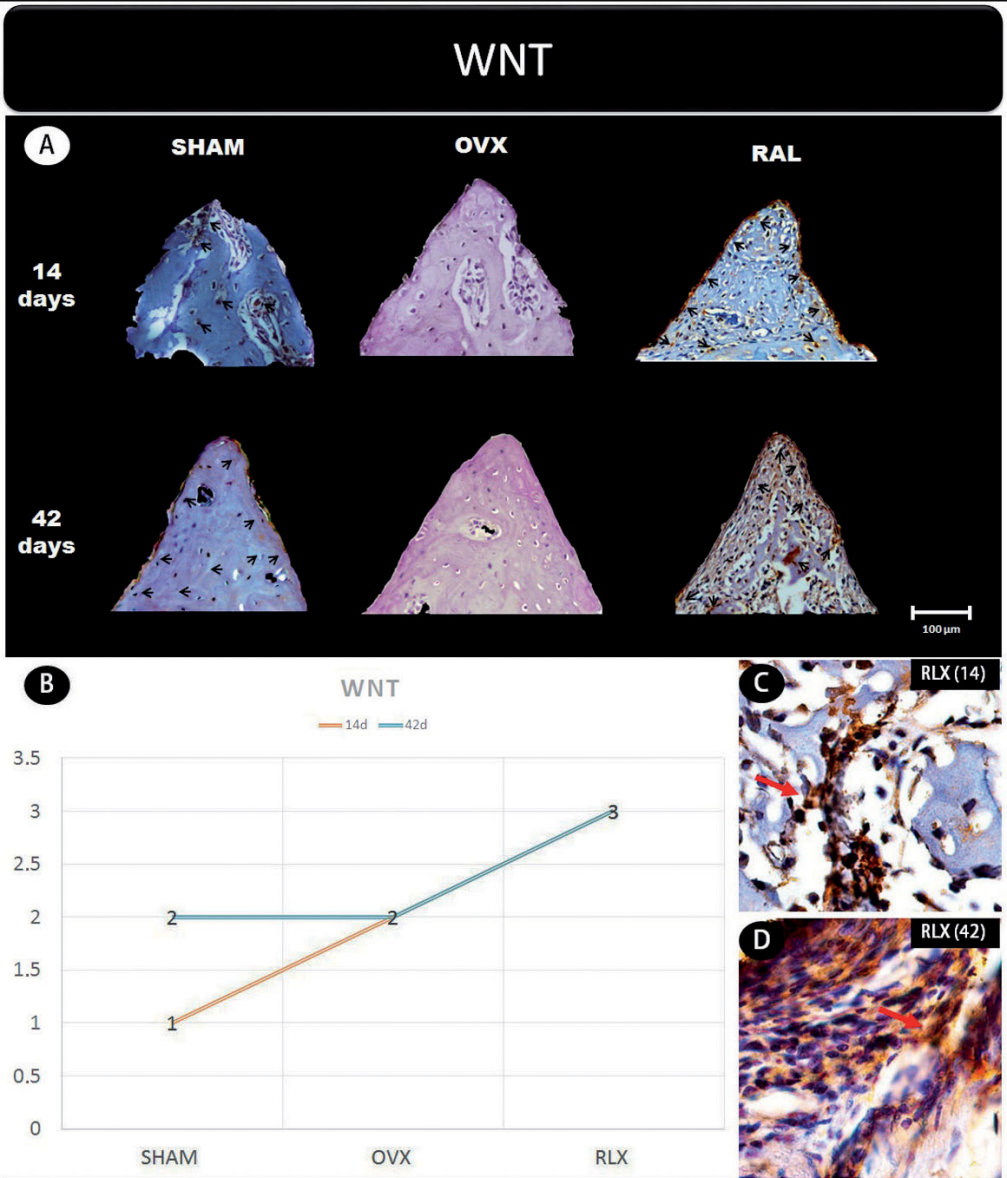
(A) Photomicrographs in a higher original objective (x40) of the different groups (SHAM, OVX, and RLX) and periods (14 and 42 days), in which is possible to observe an increased area of diaminobenzidine-stained cells (brown areas) around the peri-implant bone where the biomarker WNT were intense, represented by black arrows, denoting an improvement in the bone formation; (B) The chart shows the scores submitted to the Kappa test, in which the index was adjusted to >0.8, representing the expression of WNT among the groups and in both periods; (C and D) Photomicrographs in a higher original objective (x100) about the 14 and 42 days of peri-implant bone healing from RLX group showing an intense labeling of WNT pathway represented by red arrows

**Figure 2:**
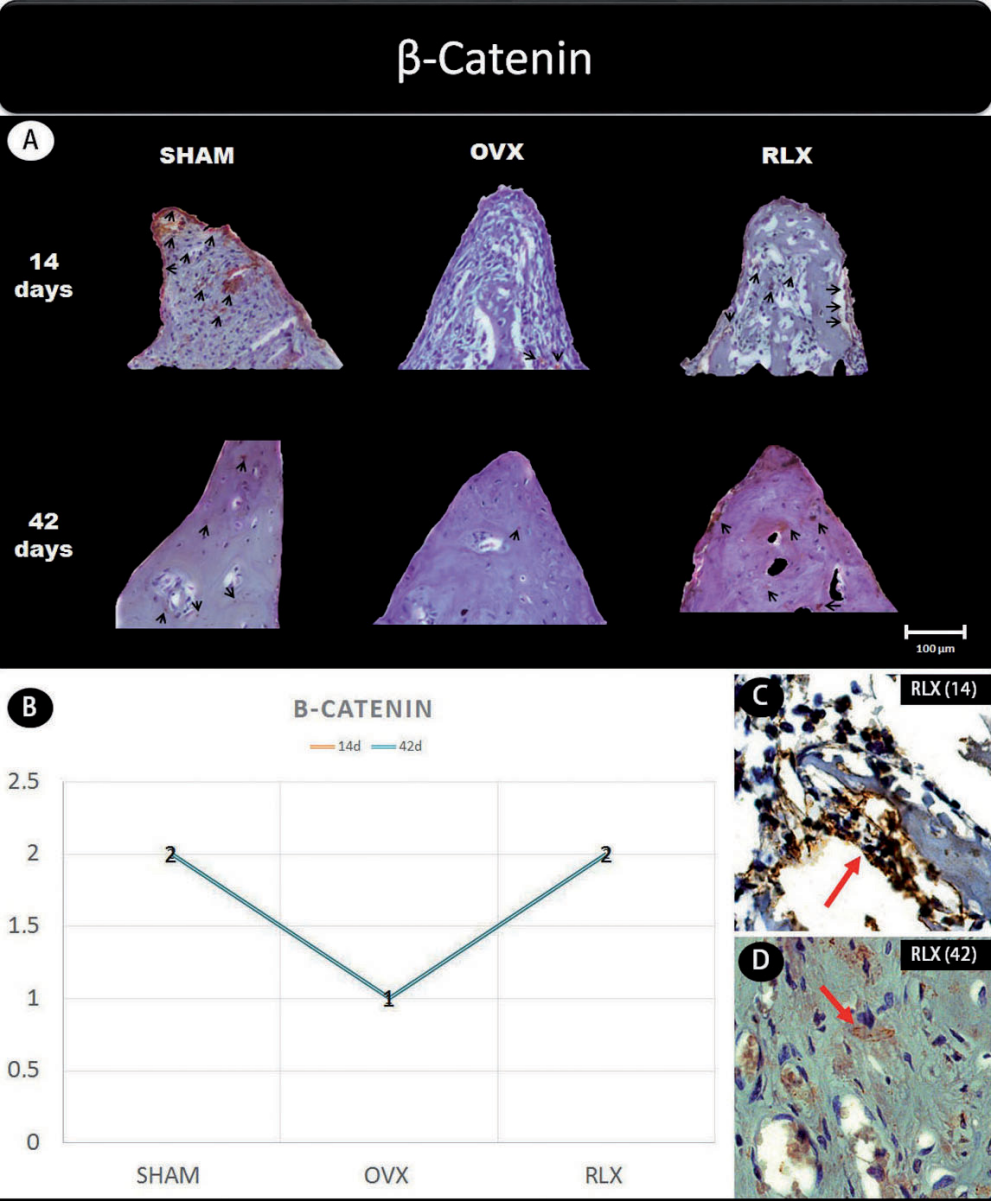
(A) Photomicrographs in a higher original objective (x40) of the different groups (SHAM, OVX, and RLX) and periods (14 and 42 days), in which is possible to observe an increased area of diaminobenzidine-stained cells (brown areas) around the peri-implant bone where the biomarker β-catenin were intense, represented by black arrows, denoting an improvement in the bone formation; (B) The chart shows the scores submitted to the Kappa test, in which the index was adjusted to >0.8, representing the expression of β-catenin among the groups and in both periods; (C and D) Photomicrographs in a higher original objective (x100) about the 14 and 42 days of peri-implant bone healing from RLX group showing a moderate labeling of the biomarker β-catenin represented by red arrows

**Figure 3:**
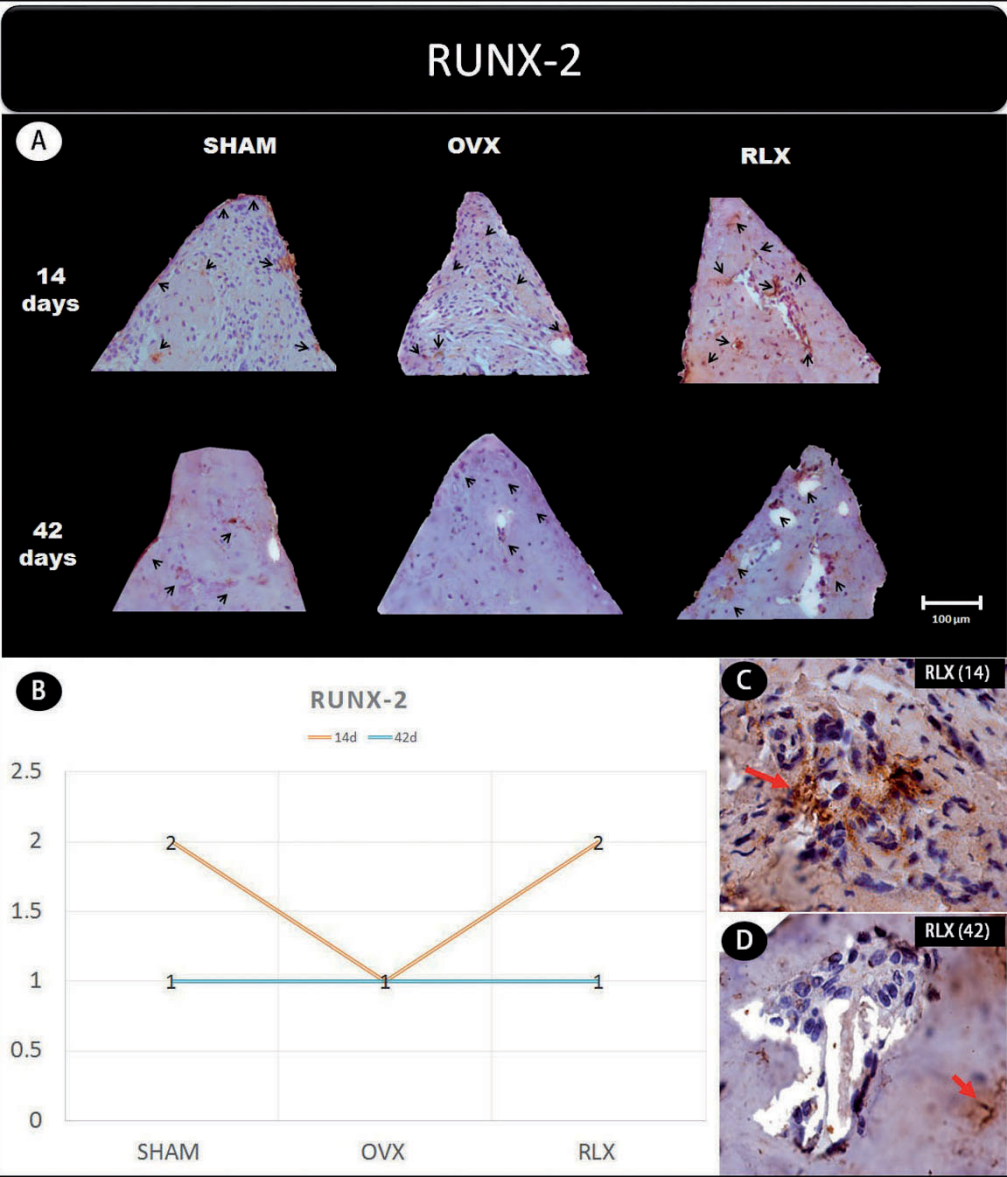
(A) Photomicrographs in a higher original objective (x40) of the different groups (SHAM, OVX, and RLX) and periods (14 and 42 days), in which is possible to observe an increased area of diaminobenzidine-stained cells (brown areas) around the peri-implant bone where the biomarker RUNX-2 were intense, represented by black arrow, denoting a greater active of the osteoblastogenesis; (B) The chart shows the scores submitted to the Kappa test, in which the index was adjusted to >0.8, representing the expression of RUNX-2 among the groups and in both periods; (C and D) Photomicrographs in a higher original objective (x100) about the 14 and 42 days of peri- implant bone healing from RLX group showing a moderate labeling for 14 days and middle labeling for 42 days of the biomarker RUNX-2 represented by red arrows

**Figure 4:**
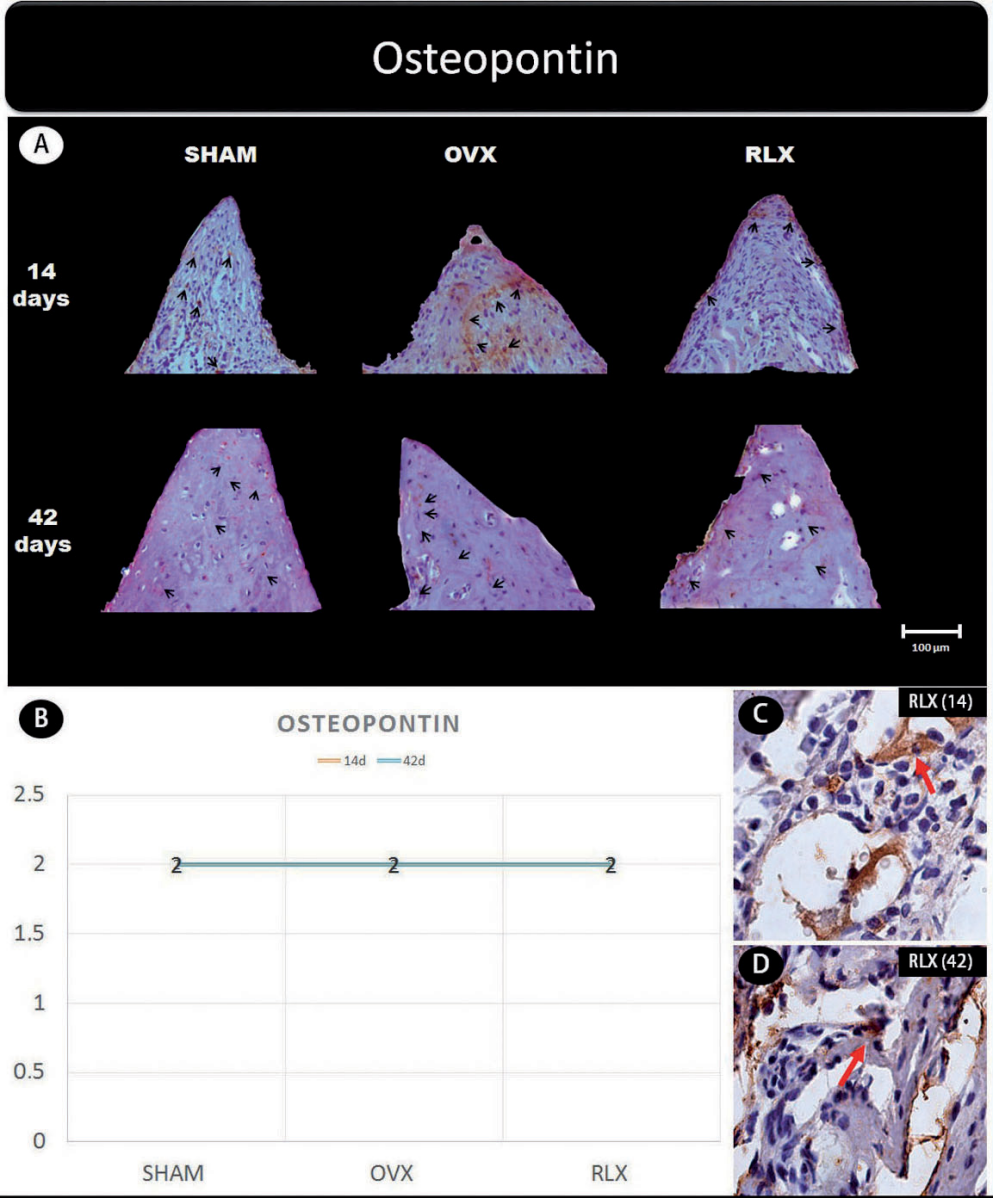
(A) Photomicrographs in a higher original objective (x40) of the different groups (SHAM, OVX, and RLX) and periods (14 and 42 days), in which is possible to observe an increased area of diaminobenzidine-stained cells (brown areas) around the peri-implant bone where the biomarker osteopontin were intense, represented by black arrows, denoting an improvement in the bone mineralization; (B) The chart shows the scores submitted to the Kappa test, in which the index was adjusted to >0.8, representing expression of osteopontin among the groups and in both periods; (C and D) Photomicrographs in a higher original objective (x100) about the 14 and 42 days of peri- implant bone healing from RLX group showing a moderate labeling of the biomarker osteopontin represented by red arrows

**Figure 5:**
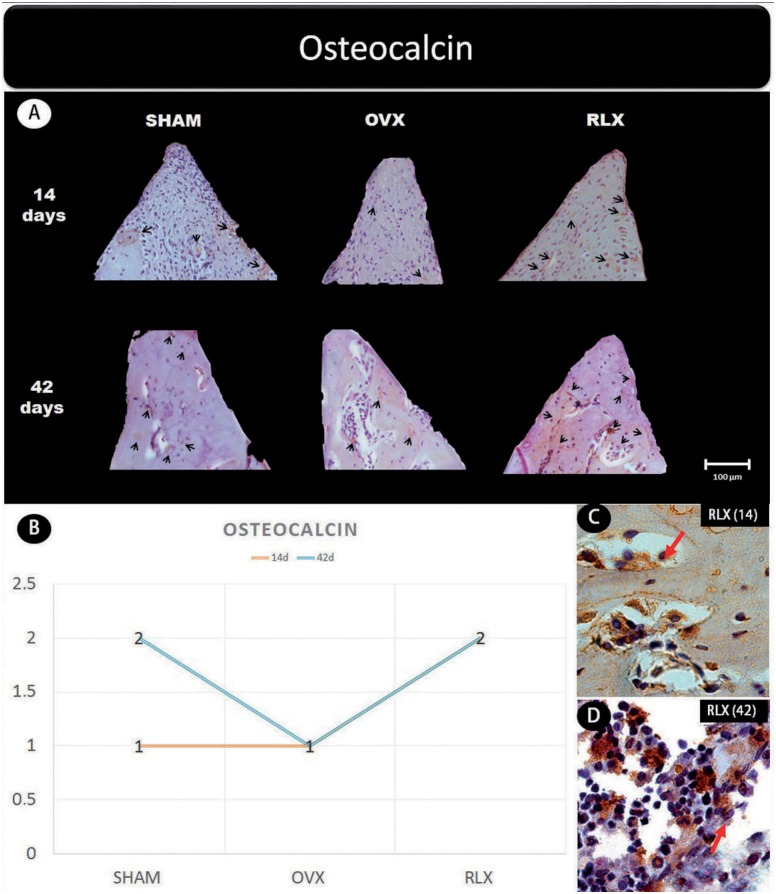
(A) Photomicrographs in a higher original objective (x40) of the different groups (SHAM, OVX, and RLX) and periods (14 and 42 days), in which is possible to observe an increased area of diaminobenzidine-stained cells (brown areas) around the peri-implant bone where the biomarker osteocalcin were intense, represented by black arrows, denoting an improvement in the bone mineralization; (B) The chart shows the scores submitted to the Kappa test, in which the index was adjusted to >0.8, representing the expression of osteocalcin among the groups and in both periods; (C and D) Photomicrographs in a higher original objective (x100) about the 14 and 42 days of peri- implant bone healing from RLX group showing a moderate labeling of the biomarker osteocalcin represented by red arrows

Representative slices and scores of immunohistochemistry may be visualized in Figures 1 to 5.

In general, the β-catenin, Wnt, and RUNX2 proteins that indicate the presence of osteogenic cell differentiation showed greater labeling for RLX groups when compared with OVX groups, regardless period of analysis (14 and 42 days postoperatively), but not for RUNX2 at 42 days, which showed mild labeling.

Concerning the mineralization proteins (osteopontin and osteocalcin) for both periods, RLX groups showed moderate labeling whereas OVX groups showed mild labeling.

### Molecular (PCR) analysis

**Figure 6:**
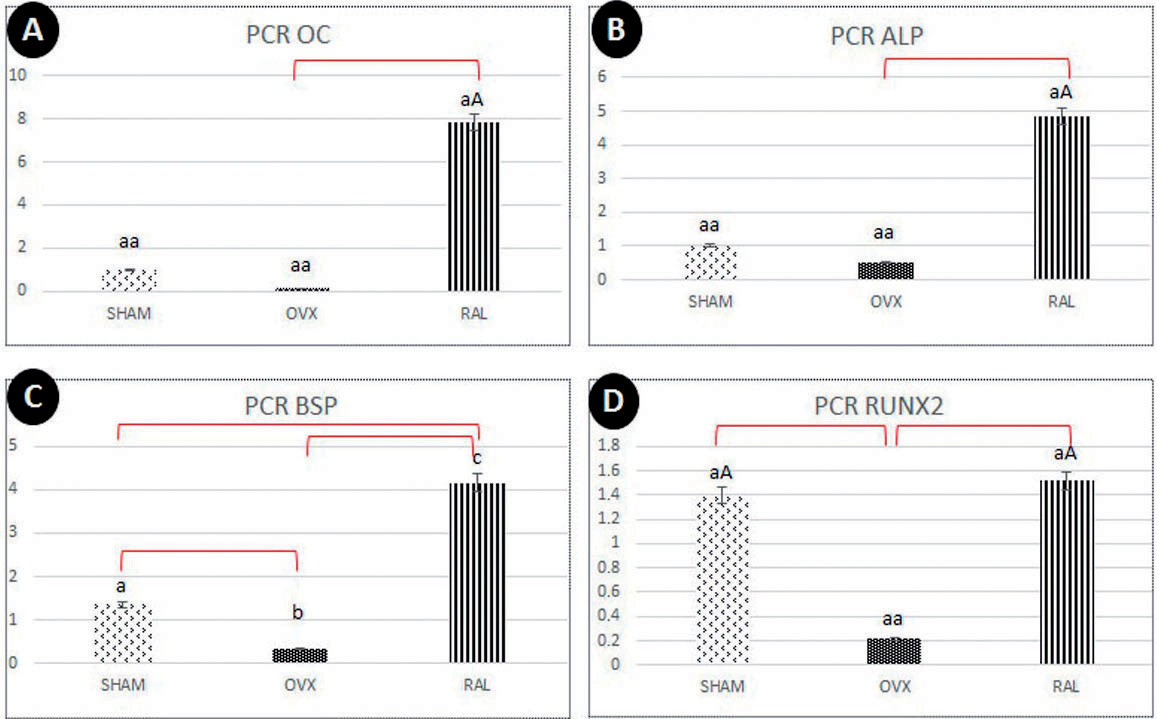
(A-D) Chart showing the highest values for RLX group regarding gene expression of osteoblastogenesis-related proteins (OC, ALP, BSP, RUNX2)

The gene expression of osteocalcin and ALP showed higher values for RLX group (P<0.05), followed by SHAM and OVX groups (P>0.05). In addition, there was no statistically significant difference between control groups (SHAM vs OVX), SHAM showed a tendency to greater values when compared to OVX (Figures 6A and B).

For BSP data, the highest expression was observed in RLX groups and the lowest expression, in OVX groups (P<0.05) (Figure 6C).

For RUNX2 data, RLX and SHAM groups showed greater values compared to OVX group (P<0.05) (Figure 6D). The interaction between SHAM and RLX groups showed similar results.

### 3D radiographic evaluation (Micro-CT)

**Figure 7:**
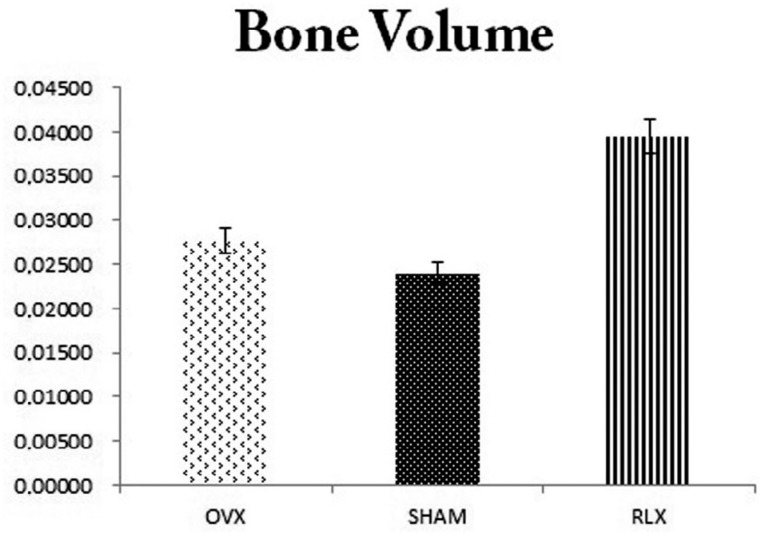
Chart with mean and standard deviation showing that RLX group tended to have higher bone volume values, followed by the OVX and SHAM groups (P>0.05)

Regarding the bone volume, we observed that RLX group tended to have higher bone volume values, followed by OVX and SHAM groups, as noted by the mean shown in the chart. However, there was no statistically significant difference between the groups (P>0.05) (Figure 7).

**Figure 8:**
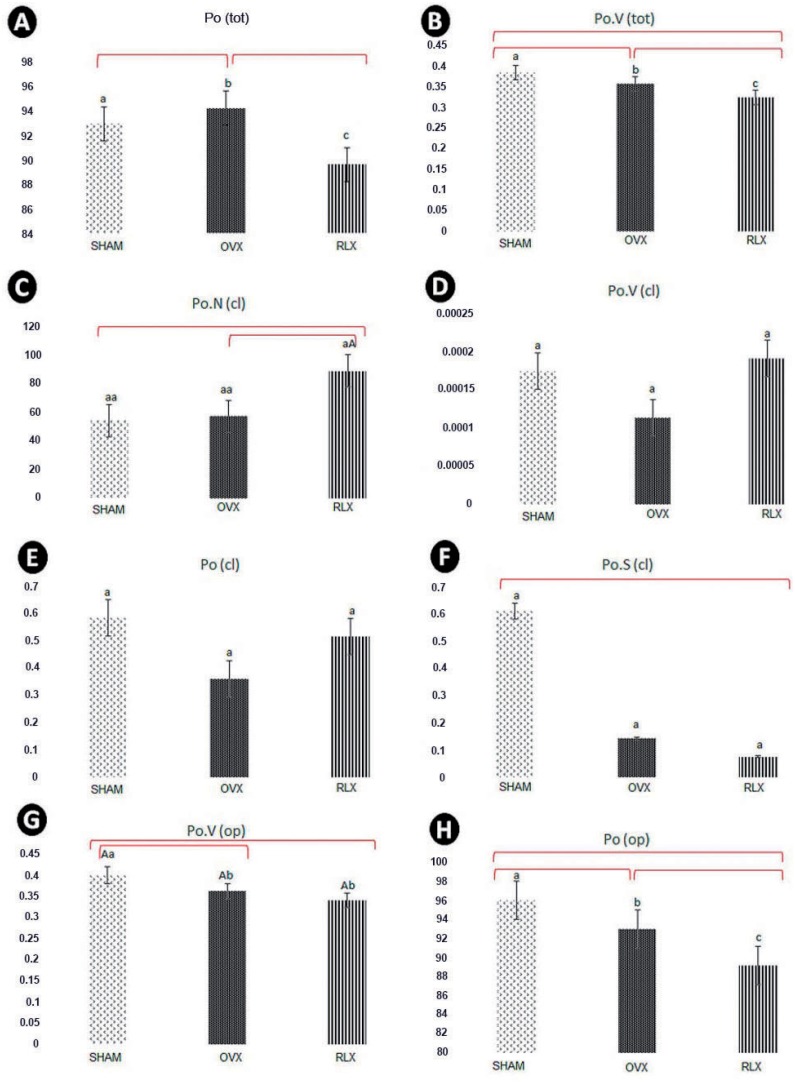
(A-H) Mean and standard deviation values of microtomographic porosity parameters in the different experimental groups (SHAM, OVX, and RLX) at 60 days after implant placement. Different letters a, b, or c, as well as difference between lower and upper-case letters show statistically significant difference between groups, according to parameters analyzed (P<0.05); same letters and lowercase letters represent similarity among groups (P>0.05) for each parameter analyzed

As for the total bone porosity, RLX group had statistically significant lower total bone porosity than OVX and SHAM groups. (P<0.05) (Figure 8A). Total volume of pore space showed higher values for SHAM group compared to OVX and RLX groups, respectively, and between OVX and RLX (P<0.05) (Figure 8B).

The microtomography parameters related to closed porosity showed higher values for number of pores (Po.N), volume of pores (Po.V), and percentage of pores (Po) in RLX and SHAM groups, whereas OVX groups showed lower results (P<0.05) (Figures 8C-E). Values of closed pores surface were higher for SHAM group followed by OVX and RLX groups (P<0.05) (Figure 8F).

Regarding the porosity open (Po.V and Po), we observed higher values for SHAM group followed by OVX and RLX groups (P<0.05) (Figures 8G and H).

**Figure 9:**
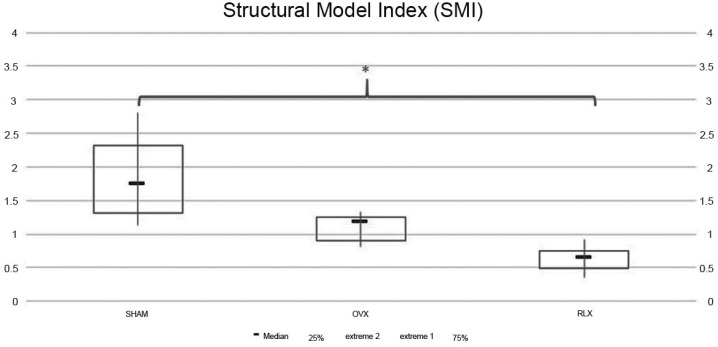
Chart with mean and standard deviation showing that RLX group had value closer to zero compared to SHAM group, which showed value closer to three (P>0.05). The asterisk represents a statistically significant difference between SHAM and RLX group

As for SMI data, RLX group showed values closer to zero, while SHAM group showed values closer to three, which means more parallel trabeculae and higher bone density (P<0.05) (Figure 9).

## Discussion

We suggested that raloxifene can stimulate the growing of pre-osteoblastics cells and promote the mineralization of bone extracellular matrix by increasing the immunolabeling of osteoblastogenesis-related proteins, such as WNT/B-catenin canonical pathway and RUNX-2. Furthermore, raloxifene increases the expression of osteocalcin, related with bone mineralization. RLX group also demonstrated to be able to enhance the value of bone volume while reduces the bone porosity. These findings provided relevant clinical impact regarding implant rehabilitation in patients with osteoporosis treated with antiresorptives drugs.

Attempting to optimize osteoporosis treatment, Ettinger, et al.^7^ (1999) conducted a randomized clinical trial (Multiple Outcomes Raloxifene Evaluation - MORE trial) that evaluated 7,705 raloxifene-treated postmenopausal women who met the World Health Organization criteria for having osteoporosis. It was observed that raloxifene was able to prevent bone loss and reduce the risk of vertebral fractures by 30-50%, but there was no significant reduction in nonvertebral fractures. However, a significant reduction in non-vertebral fractures was later found in women presenting baseline severe vertebral fractures with a risk of subsequent fractures^5^.

Experimental studies showed that raloxifene was able to optimize wound healing in rats with induced osteoporosis, relative to both hormone replacement therapy^13^ ^−^ ^15^ ^,^ ^17^ and alendronate therapy^16^ ^,^ ^18^.

Recently, the importance of canonical Wnt/β-catenin signaling in the pathogenesis of bone tissue was highlighted due to the discovery of mutations which caused a loss of function in genes related to lipoprotein receptor-related proteins (LRPs) 5/6, reducing osteoblast numbers and favoring osteoporosis onset^11^.

Some researchers believe that β-catenin accumulation in the cell nucleus may interfere in the RANK/RANK ligand/osteoprotegerin (OPG) pathway by inducing OPG expression through activation of TCF/LEF transcription factors, and thus promoting osteogenesis and inhibiting osteoclastogenesis^9^.

Another important signaling protein in this pathway is SOST, which presents itself as a Wnt inhibitor^28^ ^,^ ^30^. SOST negatively modulates Wnt by competing with it for the FZD and LRP5/6 receptors, preventing the formation of the Wnt-FZD-LRP5/6 complex, and consequently inhibiting bone formation^6^.

This study analyzed the effect of raloxifene on peri-implant healing in ovariectomized young rats and showed an intense staining of Wnt protein in the RLX group at 42 days of peri-implant repair and moderate to intense staining after 14 days. Regarding the β-catenin expression, we observed that this protein was moderately expressed at 14 days, and presented mild to moderate levels at 42 days. Thus, a possible explanation for the increased bone formation around the tibial implants in raloxifene-treated animals is that β-catenin is accumulating in osteoblastic cell nuclei, providing greater expression of OPG. These findings contrast with what was observed in the OVX group, where mild β-catenin expression and moderate Wnt expression were present at both time-points.

In an experimental study, Lin, et al.^12^ (2011) evaluated the peri-implant repair process in rat jaws by examining gene expression levels of the RUNX2 transcription factor after 3, 7, 10, and 14 days, and demonstrated that peak protein expression occurred at 14 days of repair. In addition, Stringhetta-Garcia, et al.^23^ (2016) found that raloxifene treatment for 120 days caused an increase in RUNX2 immunostaining in the tibiae of post-menopausal female rats. Accordingly, our study found an increasing immunostaining of the RUNX2 transcription factor after 14 days of peri- implant repair in the RLX group. However, at 42 days, it decreases the expression of this protein. In contrast, the OVX group showed low expression of RUNX2 at both study time-points. This finding may be explained by the fact that RUNX2 acts at the start of osteoblast differentiation, with a peak at 14 days. However, Wnt/β-catenin levels showed that raloxifene treatment stimulated the osteoblastic differentiation process, and consequently bone formation, around the tibial implants, compared to the ovariectomized animals that received no treatment, which showed low osteoblastic activity.

Despite the benefits of the drug regarding osteoblastic activity, it is important that the neo-formed bone have quality to have success in oral rehabilitation. Thus, it was evaluated the expression of osteocalcin and osteopontin as bone mineralization markers synthesized by osteoblasts, since the low expression of these proteins is associated with an increased vulnerability and fragility for long bone fractures, independent mineral density, bone volume, or porosity of the matrix^25^. Osteocalcin acts as a growth regulator of hydroxyapatite crystals, controlling the growth of the size of these crystals without prejudice to reabsorption or bone mineralization^22^. Osteopontin became more important in bone-related biomechanical functions^27^.

This study showed that there was an increasing labeling of osteocalcin and osteopontin in the RLX group both at 14 and at 42 days, in contrast to the OVX group, which showed mild expression in both periods. Osteopontin levels were found to be balanced across all groups at all time points. The positive effect of the drug is even more evident when associating immunohistochemical findings to data obtained by micro-CT, which uncovered increased bone volume in the RLX group compared to the OVX group. This is corroborated by previous studies in animals^24^, and although there was no statistically significant difference, the results allow us to state that the drug provided fundamental characteristics for the success of peri-implant repair.

Other proteins related to mineralization of bone metabolism, such as BSP and ALP, showed greater gene expression for RLX and SHAM groups compared to OVX groups. Thus, it was clear that the biological behavior of bone healing in osteoporotic rats treated with RLX was very similar to the healthy rats (SHAM). This drug was able to recover some poor quality of bone tissue observed in OVX rats with no treatment.

The amount of porosity and trabeculae geometry was analyzed in this study and demonstrates the quality of bone tissue microarchitecture. In this way, an increase of porosity parameters shows a decreasing of bone quality^1^. SMI values near to zero show the bone trabeculae like parallel plates, therefore, promoting higher bone density^1^. Thus, the results related to SMI in the RLX groups indicated improvement of quality of bone tissue. Micro-CT studies also found a decrease in bone porosity in the RLX group, whereas porosity increased in the OVX group^20^, demonstrating that treatment with alendronate was able to reduce the porosity of the cortical bone in post-menopausal women, and the positive effects of alendronate in preventing vertebral and non-vertebral fractures and increasing bone mineral density in osteoporosis was undeniable^3^ ^,^ ^29^. However, while alendronate is the most widely used drug in the world for this purpose, its prolonged use may impair bone turnover, triggering delayed bone repair and associated with oral surgeries, such as implants placement or simple teeth extractions, is closely related to development of medication- associated osteonecrosis of the jaw (MRONJ) ^21^.

Raloxifene has exerted similar benefits to those of alendronate, but it was still possible to observe the positive effects of raloxifene on peri-implant repair in ovariectomized rats, in contrast to what alendronate has shown in the long-term. Other studies regarding this phenomenon must be conducted. Although there is substantial increasingly cases of MRONJ associated with dental implants, the treatment of this condition remains unclear, being necessary to rethink if that is a good choice for osteoporosis treatment in patients who need dental implants rehabilitation. Furthermore, information on the mechanism of action of raloxifene must be clarified, since little is known about how this drug enhances the osteoblastogenesis.

The analysis periods elected for this study were based, firstly, considering the cellular expression evaluated by immunohistochemistry and PCR analysis, which are higher at 14 and 42 days postoperatively. The biomarkers WNT, β-catenin, and RUNX-2, related to osteoblastogenesis cells, are more expressive in earlier periods, such as 14 days, and the biomarkers osteopontin and osteocalcin, related to cellular mineralization, are more expressive in later periods, such as 42 days. However, at 60 days, there is no relevant cellular expression due to bone maturation. Thus, we opted to analyze the bone microstructure parameters in this period (60 days), such as bone volume and porosity, verifying if that bone would be able to receive the implants to rehabilitation.

A very important analysis to provide the safety use of the drugs would be to investigate the adverse effects. In this study, we did not perform any analysis about the systemic effect of raloxifene, such as metabolizin organs evaluation (heart, liver, brain), to show some adverse effects of this drug. Although there was no observed clinical change in the animals during the therapy with raloxifene, due to the reestablishment of metabolism induced by drug therapy, it was observed a weight gain in the animals that received raloxifene. However, further studies of our team are being perform, aiming to show not only the local effect of osteoporosis drugs (raloxifene, strontium ranelate, PTH), but the systemic effect of these drugs and whether they show any adverse effect.

## Conclusions

Regarding these findings, and despite all limitations of this study, it is possible to conclude that raloxifene treatment in ovariectomized rats provided a positive effect on tibial peri-implant repair by stimulating osteoblast differentiation concomitant to its antiresorption effect, resulting in increased bone formation. We also demonstrated that, by investigating bone matrix mineralization and microarchitecture characteristics, this newly formed bone is of high quality. We therefore believe that raloxifene must be considered in the treatment of osteoporosis, not only for its benefits in preventing fractures, but also as an adjunct in the dental implant rehabilitation process.
